# Portal Cavernoma during Pregnancy

**DOI:** 10.1155/2013/396083

**Published:** 2013-12-10

**Authors:** Fabiana Savoia, Cinzia Ferrara, Anna Sansone, Giuseppe Bifulco, Carmine Nappi, Costantino Di Carlo

**Affiliations:** Dipartimento di Neuroscienze e Scienze della Riproduzione, Università degli Studi di Napoli Federico II, Via Pansini No. 5, 80131 Napoli, Italy

## Abstract

Portal vein thrombosis (PVT) is characterized by the obstruction of the portal venous system. The venous obstruction can be partial or complete and it is caused by thrombogenic conditions (acquired or hereditary) or nonthrombotic factors. The acquired conditions include abdominal inflammation, infections, surgery, myeloproliferative disorders, obesity, oral contraceptive intake, pregnancy, and postpartum period. Occasionally, it is not possible to recognize any overt cause of PVT. During pregnancy there is an increased venous thromboembolism risk mainly in the systemic venous system and the PVT can occur, but there are no data about its exact prevalence, etiology, and outcome. The portal cavernoma is the cavernomatous transformation of the portal vein. It is a consequence of chronic PVT and occurs when myriads of collateral channels develop to bypass the occlusion. The clinical presentation includes hematemesis due to variceal bleeding, ascites or anaemia, and splenomegaly. The cavernous transformation of the portal vein is easily diagnosed by sonography. We report our case of a 32-year-old, gravida 3 para 2, pregnant woman admitted to our hospital at 13 weeks and 1 day of gestation, clinically asymptomatic. Laboratory test, ultrasound, and endoscopic evaluation were negative. After a detailed counseling, the patient decided on termination of pregnancy at 15 weeks and 1 day of gestation.

## 1. Introduction

Portal vein thrombosis (PVT) is characterized by the obstruction of the main portal vein and/or its left or right branches. The venous obstruction can be partial or complete and it is caused by thrombogenic conditions (acquired or hereditary) or nonthrombotic factors. Among the acquired conditions including abdominal inflammation, infections, surgery, myeloproliferative disorders, obesity, oral contraceptive intake, pregnancy, and postpartum period, the myeloproliferative disorders represent the most frequent etiology. Occasionally, it is not possible to recognize any overt cause of PVT. During pregnancy there is an increased venous thromboembolism risk, mainly in the systemic venous system and the portal vein thrombosis can occur, but in the literature there are no data about its exact prevalence, etiology, and outcome, and no definite guidelines for the management of this condition during pregnancy are available.

Two large studies on pregnant women with chronic PVT revealed that variceal bleeding is the most common clinical complication followed by thrombosis, abdominal pain, jaundice, and incidental splenomegaly [1, 2]. Pregnancy is characterized by a hypervolemic state that causes an increase in the portal flow, which contributes to high portal pressure that is transmitted to the upper gastrointestinal collateral veins and thus increases the risk of variceal bleeding [3].

The portal cavernoma is the cavernomatous transformation of the portal vein. The currently accepted theory is that it is a consequence of chronic PVT and occurs when myriads of collateral channels develop to bypass the occlusion. 

The clinical presentation includes hematemesis due to esophageal varices, ascites or anaemia, and splenomegaly. The cavernomatous transformation of the portal vein is easily diagnosed by sonography since gray scale and color Doppler images fail to demonstrate a normal caliber portal vein. Instead, multiple serpentine channels are seen. Color and duplex Doppler confirm the presence of portal venous type flow within those tortuous channels.

We here describe the case of a pregnant woman who was referred to our department after an incidental diagnosis of chronic PVT at 13 weeks of gestation.

The report will emphasize the clinical differential diagnosis, outcome, and management of pregnancies complicated by noncirrhotic PVT.

## 2. Case Report

The index case was a 32-year-old, gravida 3 para 2, pregnant woman. The patient was initially admitted at 11 weeks of gestation to another hospital because of a back pain and fever. A pyelonephritis diagnosis was made and antibiotic therapy with endovenous cefalexin was started. Back pain and fever were both resolved in few hours. During the hospitalization a routine abdomen scan revealed the presence of solid, hyperechoic material into a distended portal vein. The caliber of the portal vein was found increased and multiple channels were seen. Once the PVT diagnosis was confirmed with Doppler imaging, the patient was referred to a tertiary hospital. She was admitted to our department at 13 weeks and 1 day of gestation, clinically asymptomatic, without signs of hypersplenism or portal cholangiopathy. On the admission the patient was hemodynamically stable with a blood pressure of 100/50 mmHg, pulse rate of 65 bpm, and respiratory rate of 20 breaths/minute. A transabdominal ultrasound revealed a single fetus with CRL of 67 mm, corresponding to 13 weeks of gestation. According to the National Health System Guideline (Istituto Superiore di Sanità (ISS)), a first trimester screening for Down's syndrome was offered and performed with a low risk result. The nuchal translucency was 1.3 mm and the nasal bone was seen with no signs of tricuspid regurgitation and normal wave-a on the ductus venosus. A detailed and focused history taking revealed a gastric sleeve procedure performed the previous year for third degree obesity. Medical history was negative for liver disease. Laboratory investigations revealed a mild normochromic normocytic anaemia (10 mg/dL) and moderate high level of transaminases (AST 81 U/L, ALT 84 U/L) and liver function tests were otherwise unremarkable. Platelets count, prothrombin time, partial thromboplastin time and bleeding time were within the normal range. Thrombotic risk profile was negative for factor V Leiden, prothrombin gene G20210A, hyperhomocysteinemia, antithrombin III deficiency, protein C or S deficiency, and antiphospholipid antibodies. After a gastroenterological consultation the screening for viral and/or autoimmune hepatitis was performed. The autoantibodies (anti-nuclear antibody, anti-smooth muscle antibody, liver/kidney microsomal antibody, anti-soluble liver antigen, and anti-mitochondrial antibody) research was negative. Viral tests were negative for the major hepatotropic viruses (hepatitis A, hepatitis B, hepatitis C, hepatitis D, and hepatitis E) and for minor ones (herpes simplex, cytomegalovirus, Epstein-Barr virus, or Yellow fever) as well. The abdominal examination revealed a nontender, nondistended abdomen, with no pain and normal bowel sounds, of note a mild splenomegaly was found. No signs of ascites were noted. The severity of liver and other organs involvement was assessed throughout a clinical and laboratory evaluation. Abdominal ultrasound found a hepatomegaly with hypertrophy of the right lobe (anteroposterior diameter 165 mm) with nonhomogeneous hepatic echostructure and portal vein thrombosis with cavernomatous transformation. There were no signs of inflammatory abdominal foci such as appendicitis, diverticulitis, inflammatory bowel diseases, pancreatitis, cholecystitis, hepatic abscesses, and cholangitis. There was no evidence of free ascitic fluid within the abdominal cavity. Color Doppler US confirmed the cavernomatous transformation of the portal venous system in the hilar region of the liver extended to the intrahepatic system. After a discussion with gastroenterology and vascular surgery consultants, an upper digestive endoscopy was arranged and uneventfully performed. The endoscopic evaluation ruled out the presence of esophageal varices. During the hospitalization the patient was started on subcutaneous low-molecular-weight heparins 16.000 units, according to the guidelines for the treatment and prophylaxis of venous thromboembolism [4, 5]. Laboratory tests were performed weekly to monitor the activated partial thromboplastin time (PTT) and the platelets count to rule out heparin-induced thrombocytopenia and to check liver and renal functions. On the admission the patient was on endovenous antibiotic therapy with cefalexin for the pyelonephritis, which was resolved in few days. All the maternal and fetal risks related to the possible development of esophageal varices and/or other collateral circulation, ascites, and hypersplenism were exposed and clearly understood by the patient. The termination of pregnancy was offered according to the Italian law number 194/78 (article 6). A written consent form was obtained from the patient and her partner. At 15 weeks and 1 day of gestation the patient decided to have her pregnancy terminated.

## 3. Discussion

Several causes can be involved in the pathogenesis of PVT and frequently more than one coexist. Local and systemic risk factors can play a role in the pathogenesis of this condition, in the 70% and 30%, respectively [6]. The most common local thrombotic risk factor is the presence of inflammatory abdominal foci (such as appendicitis, diverticulitis, inflammatory bowel diseases, pancreatitis, cholecystitis, hepatic abscesses, and cholangitis) or a liver disease (cirrhosis or tumors). The prevalence in patients affected by a liver disease ranges from 1%, at the earlier stages, to 30% in candidates for liver transplantation. In patients with a hepatocellular carcinoma, the incidence of PVT rises to 10%–40% [7].

Other acquired conditions including infections, surgery, myeloproliferative disorders, obesity, oral contraceptive intake, pregnancy, and postpartum period can be involved in the pathogenesis. Aggarwal et al. found that an underlying hypercoagulable and prothrombotic state, as high platelets counts, was present in about 20% of the patients [2].

With the cessation of the portal venous blood flow, the liver loses about two-third of its supply. This condition is usually well tolerated, mainly due to a compensatory mechanism of venous rescue consisting of the rapid development of collaterals to bypass the obstruction. This vascular neoformation, called “cavernomatous transformation,” has been shown to form within the first 6 to 20 days after acute thrombosis of the portal vein and to be complete within 3 to 5 weeks [8, 9]. As a result the thrombosed portal vein is replaced by a network of collateral vessels, the portal cavernoma [10].

It occurs much more frequently in patients without underlying liver disease, but often leads to portal hypertension because the collateral veins are not able to adequately handle the splenic and mesenteric inflow. In cirrhosis, cavernomatous transformation of the portal vein is rare because stasis of portal venous flow prevents the formation of collateral channels in and around the portal venous thrombus. In this condition hemodynamic changes are present in hepatic and splanchnic circulation that are responsible for a partial impairment in liver function, in absence of an overt liver disease, or can precipitate a preexistent clinical status in cirrhotic patients. PVT might indirectly affect other abdominal organs, causing intestinal ischemia and infarction, or predisposition to vascular neoformation and gastrointestinal bleeding. 

In a multicentric study on maternal and fetal management and outcome, Hoekstra et al. found that in pregnant PVT patients treated with anticoagulant on an individual basis, the rate of miscarriage and preterm birth appears to be increased (38%). However, fetal and maternal outcomes are favorable for most pregnancies reaching 20 weeks of gestation [1].

Hoekstra et al. reported that the incidence of abortion, preterm deliveries, and still births in a large series of pregnancies evaluated in a tertiary centre of Northern India was of 20%, 15.4%, and 7.7%, respectively [1]. In a retrospective analysis Sumana et al. found that perinatal loss was around 25% and even lower in patients diagnosed prior to pregnancy [11].

Hoekstra et al. concluded that pregnancy should not be contraindicated in stable PVT patients [1].

In contrast to acute PVT, chronic PVT can be nearly asymptomatic as in our index case, except for the presence of esophageal varices, cutaneous collaterals, or ascites [12], with medical history apparently negative for previous trigger events or diseases. Data in the literature suggest that the most common presenting event in patients with chronic PVT is hematemesis. The exact incidence of variceal bleeding in pregnant patients with noncirrhotic portal hypertension (NCPH) is still unknown with reports ranging from an incidence of 20%–40% in a multicentric study [13] to an incidence of 34% in a prospective-retrospective analysis [1]. 

Variceal bleeding may not be as common and dreaded as previously believed. The incidence is lower in women previously diagnosed and treated [11]. A reasonable explanation is that during pregnancy there is an increased blood volume and cardiac output and most of the blood flows are within the uteroplacental circulation without affecting portal venous system and pressure.

This phenomenon is strictly time dependent and it is advisable to screen endoscopically all the patients once the diagnosis of PVT is confirmed to rule out the presence of esophageal varices [14]. Other patients can present with thrombosis, abdominal pain, and jaundice or incidental splenomegaly. Hypersplenism and the consequent pancytopenia can be present in chronic PVT [15], but if one branch of the portal vein is preserved and the portal pressure is normal (7 mmHg), they may even be absent with normal white cells, red cells, and platelets count.

The presence of an underlying liver disease influences the prognosis [15]. The overall mortality is less than 10% in PVT chronic onset, and it is higher, around 26%, in patients with malignancy or cirrhosis [16].

## 4. Conclusion

PVT is relatively uncommon in the general population and it is considered a rare pathology complicating pregnancy. Pregnancy outcome is expected to be successful in women with extra hepatic portal vein obstruction (EHPVO) if disease is diagnosed and adequately controlled prior to pregnancy [1]. In the literature there are no sufficient data regarding the management and the prognosis of the cases of portal vein thrombosis diagnosed for the first time during pregnancy.

There is no role of elective termination of pregnancy or caesarean section and these procedures should be reserved only for obstetric indications. Second stage of labor does not exacerbate upper gastrointestinal bleeding and in patients who are at high risk of variceal bleeding, it may be safely cut short by the operative vaginal delivery. Pregnancy in women with NCPH overall has a good outcome. There is no substitute for prepregnancy diagnosis and counseling and prophylactic treatment of esophageal varices before pregnancy. In our case, after a thorough literature review the patient was informed about all the maternal and fetal risks related to the possible development of esophageal varices and/or other collateral circulation, ascites, and hypersplenism. The patient was aware that her life was not threatened by this condition; nevertheless she decided to have her pregnancy terminated.
